# Textile Hemp vs. Salinity: Insights from a Targeted Gene Expression Analysis

**DOI:** 10.3390/genes8100242

**Published:** 2017-09-26

**Authors:** Gea Guerriero, Marc Behr, Jean-Francois Hausman, Sylvain Legay

**Affiliations:** Environmental Research and Innovation (ERIN) Department, Luxembourg Institute of Science and Technology (LIST), L-4362 Esch/Alzette, Luxembourg; marc.behr@list.lu (M.B.); jean-francois.hausman@list.lu (J.-F.H.); sylvain.legay@list.lu (S.L.)

**Keywords:** gene expression, hemp, salinity, bast fibers, cell wall

## Abstract

Soil salinity is a serious threat to agriculture, because it compromises biomass production and plant productivity, by negatively affecting the vegetative growth and development of plants. Fiber crops like textile hemp (*Cannabis sativa* L.) are important natural resources that provide, sustainably, both cellulosic and woody fibers for industry. In this work, the response to salinity (200 mM NaCl) of a fiber variety of hemp (Santhica 27) was studied using quantitative real-time PCR. The responses of plantlets aged 15 days were analyzed by microscopy and by measuring the changes in expression of cell wall-related genes, as well as in the general response to exogenous constraints. The results presented here show that a different response is present in the hemp hypocotyls and leaves. In the leaves, genes coding for heat shock proteins were significantly upregulated, together with a phytohormone-related transcript (ethylene-responsive factor 1 *ERF1*) and genes involved in secondary cell wall biosynthesis (cellulose synthase *CesA4*, fasciclin-like arabinogalactan proteins *FLA10* and *FLA8*). Moreover, a tendency towards upregulation was also observed in the leaves for genes involved in lignification (*4CL*, *CAD*, *PAL*); a finding that suggests growth arrest. In the hypocotyl, the genes involved in lignification did not show changes in expression, while a gene related to expansion (expansin *EXPA8*), as well as transcripts coding for calcium-dependent lipid-binding family proteins (*CALB*), were upregulated. Microscopic analyses on the hypocotyl cross sections revealed changes in the vascular tissues of salt-exposed plantlets, where the lumen of xylem vessels was reduced. The gene expression results show that a different response is present in the hemp hypocotyls and leaves. The data presented contribute to our understanding of the regulatory gene network in response to salinity in different tissues of an important fiber crop.

## 1. Introduction

During their life cycle, plants often encounter a broad spectrum of exogenous constraints caused by environmental conditions that adversely affect growth, development and, ultimately, productivity. Exogenous stressors can be biotic or abiotic in nature, whether caused by other living organisms (such as pathogens), or by deficits/chemical-physical excesses (e.g., heavy metals, salt, drought, extreme temperatures). Abiotic constraints trigger a wide range of responses which acclimatize the organism to help ensure its survival; these responses range from changes in the rate of the basal metabolism [1] to an altered expression of a specific set of genes, such as cell wall-related genes [2,3,4,5,6].

Soil salinity is a threat to agriculture; an excess of salts leads to the formation of compacted soils which reduce the cellular respiration of roots, as well as the drainage of water and substances dissolved in the organic matter [7]. Additionally, the water potential of the soil is altered, with consequent osmotic imbalances to the roots.

Many of the responses of plants to an excess of salts are related to those observed for water and osmotic stress; salinity stress is indeed due to ionic, osmotic and oxidative stresses.

One of the most immediate responses of plants to salinity is the synthesis of compatible solutes, called osmolytes, characterized by particular biochemical properties; they are highly soluble organic compounds that do not interfere with cell metabolism, even at high concentrations (recently reviewed in [8,9]). Osmolytes stabilize the hydration shell of proteins in their native conformation. The synthesis and accumulation of compatible solutes is also a strategy used by plants to lower the osmotic potential of the cytosol. Osmolytes therefore have multiple roles; they contribute to maintaining an appropriate turgor pressure in the cell, they protect the proteins against misfolding, and they mitigate the toxic effects caused by reactive oxygen species (ROS), such as protein carbamylation and lipid peroxidation.

Salinity stress induces structural changes, as, for example, seen in potato plantlets [10]; the chloroplasts were damaged and disorganized and the cell wall was thickened and ruptured. Also membrane stability is affected by salt stress, because of the accumulation of malondialdehyde, which originates from the decomposition of polyunsaturated fatty acids [11]. Like other forms of stress, salinity also causes genotoxicity, e.g., DNA damage. The hyperosmolarity caused by an excess of salts triggers the appearance of DNA fragments of double-stranded DNA (dsDNA). As a matter of fact, studies carried out on barley showed that salt stress already triggered fragmentation of DNA (called DNA laddering) after 8 h and led to cell death [12].

In the model plant thale cress, salt stress induces autophagy; mutants defective in autophagy, e.g., *ATG2* and *ATG7*, are indeed hypersensitive to salt stress [13]. Autophagy is therefore a prerequisite for tolerance to salt stress. To better understand the impact of salinity on plants, molecular and physiological studies focused on both tolerant and susceptible plants are necessary. A recent transcriptomic study on the mangrove *Avicennia officinalis*, which shows high salt tolerance, revealed that, in the roots exposed to salinity, genes involved in ethylene and auxin signaling were upregulated, while those related to abscisic acid signaling were downregulated [14]. In particular, this study identified an important role of ethylene-responsive factors (*ERF*s) in salt tolerance. Another recent study, based on proteomics, on a different plant species (a grass, *Leymus chinensis*) tolerant to salinity, confirmed the (already reported) important role of peroxidase, superoxide dismutase and catalase in the tolerance mechanism [15]. Rice is considered a salinity susceptible crop, together with wheat [8]; however, among rice varieties, there are genotypes which are more tolerant to salinity. For example, the genotype Dongdao-4 increased catalase activity, accumulated more proline and soluble sugars, and therefore showed higher tolerance to salinity [16].

Fiber crops like textile hemp and flax are important sources of raw materials for industry, as they provide, in a sustainable manner, great amounts of biomass in a relatively short time. Soil salinity can negatively affect the production of plant biomass and therefore has a strong impact on fiber crop productivity. Some studies are available in the literature on the effects that salinity exposure has on fiber crops. For example, in flax, NaCl exposure (50 mM NaCl) triggered the increased expression (log_2_FC as compared to control >6.8) of a *NAC47* gene, together with a β-glucosidase 17, a dehydrin and a cytochrome *CYP82C4* [17]. More recently, the response to salinity (500 mM NaCl) was compared in the leaves of a seed vs. textile hemp variety using RNA sequencing (RNA-Seq) [18]: this study demonstrated that the fiber variety, after salt stress, showed an enrichment in genes belonging to the spliceosome ontology and amino acid metabolism, while in the seed variety there was a predominance of genes related to fatty acid and amino acid metabolisms, as well as endoplasmic reticulum protein processing pathway. In the same work, 220 co-upregulated differentially expressed genes were identified, among which several transcription factors belonging to the MYB, NAC, GATA and HSF families.

Hemp was previously shown to be a valid model for carrying out molecular investigation focusing on the cell walls; the hypocotyl shows a temporal separation of elongation and secondary growth [19], while adult plants have a basipetal gradient of lignification and of bast fiber developmental stages [20].

In the present study, the effects of NaCl exposure on a fiber variety of *Cannabis sativa* (cv. Santhica 27) were analyzed by means of targeted gene expression. The goal was to elucidate the effects that salt exposure triggers in hemp plantlets aged 15 days (a time-point characterized by cessation of bast fiber primary growth and the start of cell wall thickening [19]), by monitoring genes involved in cell wall biosynthesis, as well as in the general response of plants to exogenous constraints in both leaves and hypocotyls. We provide a description of the events associated to NaCl exposure using a cell wall perspective, which has so far not been done to our knowledge.

## 2. Materials and Methods

### 2.1. Experimental Set-Up, Plant Material and Growth Conditions

The hemp monoecious fiber variety Santhica 27 was used in this study. Plantlets were grown in pots (size: 10 × 10 × 12 cm) filled with a mixture of compost/sand (1:1 *w*/*w*) in controlled conditions, as previously described [19]. Salt treatment was performed with 200 mM NaCl (60 mL/pot) which was supplied to plants twice a week. Control plants were supplemented with 60 mL water. The concentration used corresponds to the window of values considered as a high level of salinity [21]. The application was not step-wise, therefore the set-up only partly mirrors the actual field growing conditions where salt shock is prevented by a gradual increase of salts [4]. This choice was motivated by the willingness to trigger the genetic response to the osmotic component of the salt shock [21]. The first application of NaCl was carried out on plants aged 4 days; the treatment lasted until the plantlets were 15 days old. Three biological replicates were sampled, each consisting of a pool of 8–10 plantlets. The hypocotyls were sampled as previously described [19], the leaves (including the first pair of true leaves) were quickly removed and frozen in liquid nitrogen. The diameters of the hypocotyls grown under control condition and NaCl exposure were measured (*n* = 28 for the control and *n* = 32 for salt-exposed plants) using a digital caliper.

### 2.2. Soil Electric Conductivity and pH Measurement

After plant sampling, soil samples from each biological replicate and treatment were collected (*n* = 3 for each treatment). For each sample, the soil was homogenized and dried at 105 °C for 72 h. Five grams of each sample were collected in a 50 mL Falcon tube supplemented by 25 mL of Milli-Q water (Merck, Kenilworth, NJ, USA). After a thorough mixing, samples were agitated on a rolling table for one hour. The electric conductivity (ECe) and pH were measured using an inoLab Multi 9430 IDS (WTW, Weilheim, Germany). The conductivity and pH of the soils were measured as previously described [22].

### 2.3. Toluidine Blue Staining and Immunohistochemistry

Fifteen-day-old hypocotyls were fixed overnight at 4 °C in 4% paraformaldehyde (Sigma-Aldrich, Steinheim, Germany) in phosphate buffered saline (PBS). After washing, sections of 5 mm were embedded in 5% agarose. Cross sections of 100 µM were cut using a vibratome (Leica Biosystems, Nussloch, Germany) and were stained with toluidine blue (Sigma-Aldrich), or used for cellulose immunodetection. The cellulose-binding module CBM3a probe (PlantProbes, Leeds, UK) specific for crystalline cellulose was diluted to 10 µg/mL in milk protein/PBS (MP/PBS, 5% *w*/*v*), incubated in mouse anti-His monoclonal antibody (1% in MP/PBS, Sigma-Aldrich) and finally incubated in 2% anti-mouse IgG coupled to fluorescein isothiocyanate (FITC) (Sigma-Aldrich) in MP/PBS. CBM3a incubation lasted for 1.5 h. Anti-His and IgG-FITC incubation lasted 1 h. Between each step, three washes of 5 min each with PBS were performed. The slides were mounted in 50% glycerol (Sigma-Aldrich) and observed with a confocal microscope LSM 510 Meta (Zeiss, Jena, Germany) with the following setting: excitation at 488 nm, filter HFT 488/594 and emission recorded with LP 505, as previously reported [19].

### 2.4. RNA Extraction and Reverse Transcription-quantitative PCR (RT-qPCR)

Total RNA was extracted and checked as previously described [12]. The RNA integrity numbers (RINs) were >7.5 for all the samples studied. One microgram of total RNA was retrotranscribed into complementary DNA (cDNA) using the ProtoScript II RTase (NEB, Leiden, The Netherlands) and random hexamers, as previously described [19,23].

The expression was calculated using 3 reference genes (*eTIF4E*, *TIP41* and *RAN* which geNORM (implemented in qBasePLUS, Biogazelle, Ghent, Belgium) identified as sufficient for data normalization and chosen for the previously reported candidates [23]). Statistical analyses were carried out using IBM SPSS Statistics v19 (IBM SPSS, Chicago, IL, USA) (*t*-test for independent samples).

### 2.5. Primer Design

Primers were designed using Primer3Plus [24] and verified with the OligoAnalyzer 3.1 tool from Integrated DNA Technologies (Leuven, Belgium) (http://eu.idtdna.com/calc/analyzer). Primer efficiencies were calculated via RT-qPCR using a serial five-fold dilution of cDNA (25, 5, 1, 0.2, 0.04, 0.008 ng/µL). The primer sequences, amplification efficiencies and *R*^2^ have either been previously published (*eTIF4E*, *TIP41*, *F-BOX*, *RAN*, *PAL*, *CAD*, *4CL*, *FLA1*-*3*-*6*-*8*-*10*, *EXPA8*, *CesA4*-*7*-*8* [19,23]), or are indicated in Table 1. The identification numbers (IDs) of the genes actin depolymerizing factor 5 *ADF5*, ethylene response factor *ERF1*, auxin-responsive protein *IAA11*, calcium-dependent lipid-binding family protein isoform 2 *CALB2*, calcium-dependent lipid-binding family protein isoforms 4 *CALB4-1*,*-2*,*-3*, heat shock protein 70 *HSP70-1* and *-2*, heat shock protein *HSP81.4*, and gibberellin receptor 1b *GIBB-REC*, histidine kinase 4-like *HK4* have been previously described [18].

## 3. Results and Discussion

### 3.1. Phenotype of Hemp Plantlets Exposed to NaCl

pH and electric conductivity measurements suggest that the soil can be considered as moderately saline (4 < ECe < 9, pH < 8.5) [25]. Indeed, values of the electric conductivity were 0.813 ± 0.14 mS cm^−1^ for the control and 5.1 ± 0.44 mS cm^−1^ for the NaCl-treatment (*p*-value = 0.00019); a slight but significant increase in the pH was also observed (5.1 ± 0.046 for the control and 5.47 ± 0.060, *p*-value = 0.0073).

**Figure 1:**
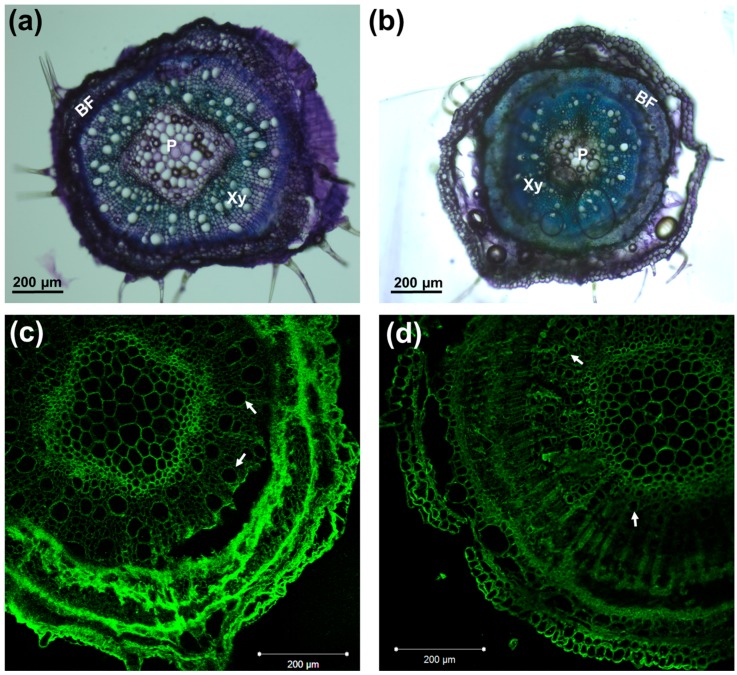
Toluidine blue staining of cross sections of control (**a**) and salt-exposed (**b**) hypocotyls; CBM3a immunodetection in control (**c**) and salt-exposed plants (**d**). Arrows indicate xylem vessels. BF: bast fibers; Xy: xylem; P: pith.

The diameters of the control and salt-exposed hemp hypocotyls were measured with a digital caliper and the results showed a statistically significant reduction on the hypocotyl thickness (1.31 ± 0.13 mm for the control and 1.06 ± 0.11 mm for salt exposure; *p*-value = 1.24 × 10^−11^. Salt stress is known to affect root tips by impairing growth via modification of cell adherence and tissue continuity (due to plasmolysis) [26]. In the hemp shoots, more frequent cortical tissue detachments were observed in the sections of salinity-exposed plantlets (Figure 1b), a finding suggesting that salinity may impair tissue continuity in aerial tissues, in a manner analogous to what was previously reported in root tips [26]. It should, however, be noted that, despite the high NaCl concentration used, no visible signs of shock in the aerial parts of hemp plantlets could be noticed after the first application; while we cannot exclude the short-term (within hours) differential regulation of genes related to the osmotic component of salt shock, the aerial tissues appeared, phenotypically, as turgid as control plants, and no discernible discoloration could be noticed, either. Hemp is known to be a robust crop vs. exogenous constraints; nevertheless, different varieties showing varying susceptibility to salinity have already been studied in the literature [18]. Although further analyses are necessary to fully understand the susceptibility to salinity of the cultivar here studied, i.e., Santhica 27, we can claim that noticeable effects (growth arrest, loss of turgidity in the leaves) started to be clear only after 2 weeks of treatment. Microscopic observations carried out with the toluidine blue staining and crystalline cellulose detection methods using the CBM3a antibody revealed the presence of smaller xylem vessels in the NaCl-exposed hemp plantlets (Figure 1). The occurrence of smaller xylem vessels is a characteristic previously observed in the xylem of other species, e.g., poplar [27]; the smaller lumen is a mechanism of resistance to cavitation, whose possibility to occur increases because of salinity.

### 3.2. Gene Expression Pattern in the Leaves

The gene expression analysis performed differentiated the leaves and hypocotyls, because the two tissues provide different physiological information: while leaves provide data on the prominent photosynthetic tissues, the hypocotyls give more focused information concerning secondary growth and bast fiber formation [19] under salt exposure.

The genes targeted are divided into 4 groups: (1) lignin-related (phenylalanine ammonia lyase *PAL*, 4-coumarate:CoA ligase *4CL*, cinnamyl alcohol dehydrogenase *CAD*); (2) secondary cell wall-related (cellulose synthases *CesA4*, *CesA7*, *CesA8*, gene encoding the fasciclin-like arabinogalactan protein *FLA3*); (3) primary cell wall-related (expansin *EXPA8*, *FLA1*-*6*-*8*-*10*); and (4) stress-related (*ADF5*, *ERF1*, *IAA11*, *CALB2*, *CALB4-1*,*-2*,*-3*, *HSP70-1* and *-2*, *HSP81.4*, *GIBB-REC*, and *HK4*).

**Figure 2:**
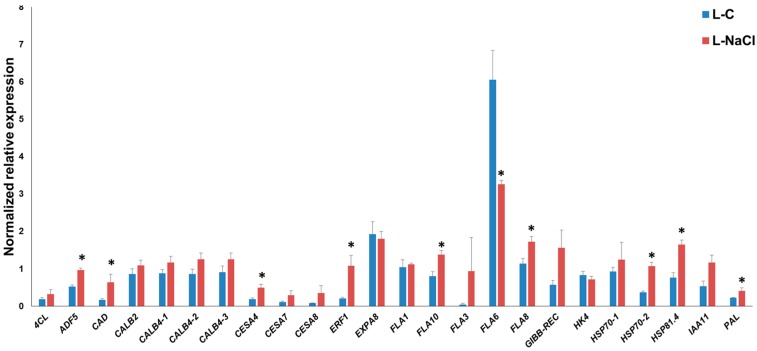
Reverse Transcription-quantitative PCR (RT-qPCR) of hemp leaves under control (L-C) and NaCl exposure (L-NaCl). The asterisks (*) indicates statistically significant differences calculated with the Student’s *t*-test (*p* < 0.05).

As can be seen in Figure 2, in the leaves, specific *FLA* genes showed differential expression under salt exposure; *FLA8* and *FLA10* showed a statistically significant increase in expression, while *FLA6* was downregulated. It is known that *FLA* genes show differences in expression in plants after abiotic stresses; for example, in poplar, the *FLA* genes of class III were found to be responsive to salinity [28]; likewise, specific *FLA*s in rice (*OsFLA23* [29]) and wheat (*TaFLA12* [30]) were induced upon salt treatment. In this respect, it is known that in *A. thaliana FLA4*/*SOS5* (salt overly sensitive 5) is important for normal root cell development under elevated salinity [31]; therefore, *FLA*s do play an important physiological role (linked to the cell wall-plasma membrane continuum) in response to abiotic constraints. We recently showed that *FLA6* belongs to Class C (corresponding to Class III described by [29]), and that its expression is higher in both leaves and roots [32]. The decreased expression of *FLA6* in hemp may indicate a role under salt exposure.

The increased expression pattern of hemp *FLA8* and *FLA10* suggests a different role with respect to *FLA6* in the response of hemp leaves to NaCl treatment: like *FLA6*, *FLA8* and *FLA10* were also shown to be highly expressed in the leaves [32].

Soil salinity can cause secondary stresses in plants; ROS, such as hydrogen peroxide, coupled to the activity of peroxidases, may cross-link the cell wall and stiffen it [33]. Cell wall stiffening may be consolidated by the deposition of lignin [34] via the activation of genes involved in the phenylpropanoid pathway. The secondary cell wall *CesA4* showed a statistically significant increase in expression in salt-exposed leaves, and this is accompanied by a tendency towards upregulation for the other secondary cell wall *CesA*s, *CesA7* and *CesA8*, together with the lignification-related genes *4CL*, *CAD* and *PAL* (for these two genes the differential expressions are significant) (Figure 2). These data indicate that in the leaves of NaCl-exposed hemp plantlets, the genes involved in secondary cell wall synthesis and lignification are induced; this likely induces stiffening of the cell walls and results in a reduced growth, as previously observed in salt-sensitive maize leaves [35]. The upregulation of genes involved in secondary cell wall formation and lignification was previously reported on NaCl-exposed leaves of alfalfa [3].

Among the stress-related genes, *ADF5*, *ERF1*, *HSP70-2* and *HSP81.4* were significantly induced upon salt exposure: these data show that an active response to the abiotic stressor takes place via the modulation of genes involved in cytoskeleton remodeling (*ADF5*, which regulates F-actin organization/dynamics and thereby controls growth of plant cells under exogenous constraints [36]), ethylene response (*ERF1* transcription factor, which was shown to play a positive role under salt, drought and heat stress in thale cress [37]) and protein folding (*HSP70* and *HSP81.4*, notably in a proteomics study, HSP70 increased after NaCl application in hemp [38]).

### 3.3. Gene Expression Pattern in the Hypocotyls

**Figure 3:**
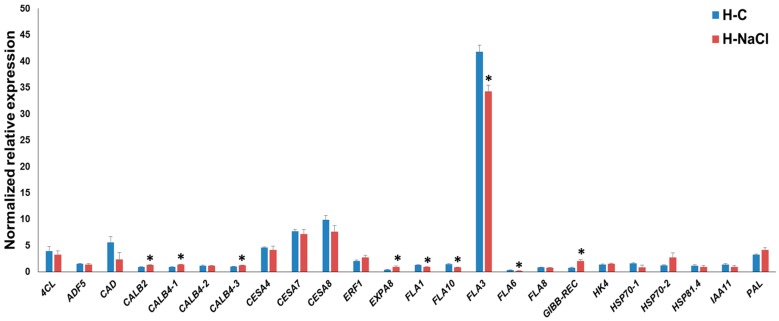
RT-qPCR of hemp hypocotyls under control (H-C) and NaCl exposure (H-NaCl). The asterisks (*) indicates statistically significant differences calculated with the Student’s *t*-test (*p* < 0.05).

In the hypocotyls, the *FLA* genes *FLA1*-*3*-*6*-*10* were significantly downregulated under salt exposure (Figure 3). We previously showed that *FLA3* is upregulated at later stages of hypocotyl development [32]; hence, we may infer that this gene is involved in processes related to the hypocotyl thickening. Its downregulation upon NaCl treatment in the hypocotyls (and it should be noted that in the leaves *FLA3* shows an increase in expression instead, as shown in Figure 2) indicates that the hypocotyl tissue responds by activating the gene program associated to cell expansion and growth. In support of this hypothesis, *EXPA8* was upregulated, and the secondary *CesA*s, as well as *4CL* and *CAD*, displayed a decreased expression trend under salt exposure (although this was not statistically significant). These results point to the existence of a different regulatory network under NaCl exposure in the leaves and the hypocotyls of young hemp plants.

Among the stress-related genes, *CALB2*, *CALB4-1* and *CALB4-3* show a statistically significant increase in expression upon NaCl treatment; calcium-dependent lipid binding proteins have been proposed to partake in intracellular signaling upon stress [39]; however, many of these genes await functional characterization in plants. Recently, a CALB protein, AtCLB, was shown to mediate the response to abiotic stresses via ceramide signaling (which triggers programmed cell death) and, interestingly, to be able to bind DNA [39]. It will be interesting to functionally study the role of these *CALB* genes in hemp under stress. The gene *GIBB-REC* also shows a statistically significant increase in expression, a finding which indicates an involvement of phytohormone-related signal transduction in the hemp hypocotyls in response to NaCl application.

## 4. Conclusions

Our study shows the existence of a different response mechanism to NaCl exposure in photosynthetic tissues and hypocotyls of textile hemp plantlets. While in the leaves upregulation of genes involved in secondary cell wall/lignin biosynthesis was induced, the hypocotyls showed an increase in expression of those genes involved in cell elongation. The response observed in the hypocotyl is important, as it undergoes secondary growth; therefore, the mechanisms upregulated in response to NaCl exposure reflect the gene network regulating bast fiber development in response to abiotic stressors. Bast fibers are cells fulfilling an important mechanical role and they are characterized by a noteworthy length; therefore, cell elongation is one of the most crucial processes that needs to be preserved upon abiotic constraints in fiber crops. It will be interesting to investigate, in the future, whether the same response is observed in the hypocotyls of those fiber crops producing xylan-type bast fibers (i.e., jute, kenaf), to understand whether a gene regulatory network preserving cell elongation under abiotic constraints is shared by both gelatinous and xylan-type bast fibers.

## Figures and Tables

**Table 1:** List of primers used in the study, with details of the amplicon size and amplification efficiencies.

Primer Name	Sequence (5′ → 3′)	Amplicon Size (bp)	Amplification Efficiency (%)
**IAA11 Fwd**	GTGGCCTCCAATCAGAACTTAC	79	110.7
**IAA11 Rev**	GCTGAATCCTTCATTGAGTGG		
**ADF5 Fwd**	TGGATGTGATTCAGGACAGG	116	105.5
**ADF5 Rev**	TGGATAAAAGCATAGCCCTTG		
**ERF1 Fwd**	TACTTCAATGGCAGCAGCAC	96	105.3
**ERF1 Rev**	TTTGGTGGTGGGTCGTTTAG		
**CALB2 Fwd**	GCTTCCTCCAAGTTTTCGTG	128	93.8
**CALB2 Rev**	CAACCACAACCGTCGATATG		
**CALB4-1 Fwd**	TGTTCTTGCTCATCCTGCTC	114	92.8
**CALB4-1 Rev**	GCTTCAAGGGTTGCTGATTC		
**CALB4-2 Fwd**	GGCTGGTAGGCAAGTTTTTG	90	94.2
**CALB4-2 Rev**	TGAAGCTCCAATCCCCTATG		
**CALB4-3 Fwd**	ATCTGGGCGAGATATTGCTG	85	95.7
**CALB4-3 Rev**	CGAGTCATAACTTGGCACAAC		
**HSP70-1 Fwd**	TCAACCATTTCGTCCAGGAG	98	91.6
**HSP70-1 Rev**	CGCTCTCTCACAAGATGTTCTC		
**HSP70-2 Fwd**	GGGATTCTGAATGTGTCTGC	92	104.1
**HSP70-2 Rev**	TCTTCCTTGCTCAACCTTCC		
**HSP81.4 Fwd**	AGGAGTTCATGGAGGCATTG	94	92.2
**HSP81.4 Rev**	TTCTCGGCAACAAGGTAAGC		
**GibbRec Fwd**	TAATCTTCTTCGGCGGTCTG	102	102.6
**GibbRec Rev**	GAGAAAACCCCATCAACAGG		
**HK4 Fwd**	TGCTGAGGTGGGTTTTTCTC	139	93.5
**HK4 Rev**	TTGTGCTGCATCTTCAGACC		
